# Psychosocial profiles of bystander resuscitation among Macao residents: a latent profile analysis

**DOI:** 10.3389/fpubh.2026.1867128

**Published:** 2026-08-07

**Authors:** Ka Kit Wong, Sok Leng Che, Aimei Mao, Ka Kei Chao

**Affiliations:** 1 Nursing and Health Study Centre, Kiang Wu Nursing College of Macau, Macao, Macao SAR, China; 2 Nursing and Health Education Research Centre, Kiang Wu Nursing College of Macau, Macao, Macao SAR, China; 3 Department of Education, Kiang Wu Nursing College of Macau, Macao, Macao SAR, China

**Keywords:** attitude, automated external defibrillator, bystander resuscitation, cardiopulmonary resuscitation, latent profile analysis, perceived behavioral control, public access defibrillation, subjective norm

## Abstract

**Introduction:**

Bystander cardiopulmonary resuscitation (BCPR) and public access defibrillation (PAD) are critical to improving survival after out-of-hospital cardiac arrest (OHCA). However, the rates of BCPR and PAD remain suboptimal in many Asian regions. Although psychosocial determinants of bystander resuscitation have been widely examined using the Theory of Planned Behavior (TPB), most studies adopt variable‑centered approaches that might underestimate individual‑level heterogeneity. This study aimed to identify psychosocial profiles related to bystander resuscitation among Macao residents using a person‑centered analytic approach and to examine socio‑demographic factors associated with profile membership.

**Methods:**

A cross-sectional online survey based on TPB constructs recruited 579 community-dwelling adults through convenience and snowball sampling. Latent profile analysis was performed to identify specific psychosocial profiles. Multinomial logistic regression was used to examine the association between socio-demographic factors and profile membership.

**Results:**

Latent profile analysis revealed four distinct psychosocial profiles: reluctant, socially inhibited, technically constrained, and proactive. Willingness to provide bystander resuscitation varied significantly across profiles, with the highest in the proactive group and lowest in the reluctant group. Socially inhibited individuals showed high perceived behavioral control (PBC) despite low subjective norms, whereas technically constrained individuals demonstrated low PBC despite favorable attitudes and subjective norms. Multinomial logistic regression indicated that lack of prior resuscitation training and older age were associated with lower odds of belonging to the proactive group.

**Conclusion:**

Findings underscore the central role of PBC and highlight the value of profile‑ and age‑specific training strategies to enhance community bystander resuscitation willingness.

## 1 Introduction

Out-of-hospital cardiac arrest (OHCA) has long been a global public health issue. Globally, the annual incidence of OHCA is estimated to range from 30.0 to 97.1 per 100,000 persons. However, the overall rate of survival to admission or 1 month was 3.1–20.4% (1), whereas a lower rate of survival to discharge (1.0–16.7%) was observed in low-, middle-income, and developing Asian countries (2). Immediately providing bystander cardiopulmonary resuscitation (BCPR) and public access defibrillation (PAD), also known as using an automatic external defibrillator (AED) in the community, play a crucial role in OHCA prognosis, significantly improving survival rates and neurological outcomes (1, 3–5). Although the proportion of providing BCPR increases over time and reaches a plateau of 50–60% (6), the proportion of providing PAD was less than 1% across Asian countries/regions according to the Pan Asian Resuscitation Outcomes Study (PAROS) (7). In China, only 20 and <0.1% of OHCA patients received BCPR and PAD, respectively (8).

The willingness to provide BCPR and PAD serves as a proxy indicator for estimating actual bystander response rates during OHCA events, especially in settings where comprehensive emergency medical services (EMS) registries are lacking. Numerous studies have identified a wide range of psychosocial factors influencing the willingness to provide BCPR and PAD. Daud et al. (9) found six key factors, including the socio-demographics (male, younger age, higher level of education, married, employed), training (having participated in CPR and AED education), attitudes and perception (more positive), self-efficacy (more confident), and legal obligation (with protection). Others reported that profession (healthcare provider), more knowledge and skill level, willingness to participate in training, interest in first-aid, having first-aid experience, assisted with AED voice prompt, and having friends with heart diseases, also positively influenced the willingness to provide BCPR and PAD (10–12). In contrast, fear of legal issues and concern about causing harm to patients (13), fear of disease transmission, blood exposure, and perceived unsafety (14), anxiety about negative outcomes (15), lack of community cohesion (16), as well as language and communication challenges (17) were identified as the psychosocial barriers toward BCPR and PAD willingness.

In recent years, the Theory of Planned Behavior (TPB) framework has been frequently employed to examine factors influencing willingness to provide BCPR and PAD (18–20). According to TPB, intention is considered the most significant predictor of actual behavior and is determined by three components: attitude, subjective norms, and perceived behavioral control toward the behaviors (21). From the perspective of bystander responses to OHCA, individuals’ willingness to provide BCPR and PAD (intention) is influenced by their beliefs about the effectiveness of BCPR and PAD (attitude), perceived social expectations (subjective norms), and their confidence or self-efficacy in providing BCPR and PAD (perceived behavioral control).

While the TPB provides a theoretical framework for examining bystander behaviors in emergency situations, previous studies have primarily focused on independent factors associated with willingness to provide BCPR and PAD using a variable-centered approach. Consequently, person-centered factors and their underlying influences may be largely underestimated, especially in Asian countries and regions where BCPR and PAD rates remain relatively low. Therefore, the purpose of this study was to examine specific psychosocial profiles of bystander resuscitation among community-dwelling individuals. A further aim is to identify the characteristics of individuals across different profiles, thereby providing a basis for tailored interventions to enhance the willingness to provide BCPR and PAD during OHCA.

## 2 Materials and methods

### 2.1 Study design and recruitment of participants

This was a cross-sectional study that used an online questionnaire to recruit residents in Macao, China. Convenience sampling combined with the snowball strategy was adopted. The residents aged 18 or older were 466,800 at the end of 2024, accounting for 82.1% total population of Macao (22). The required sample size was calculated using the formula $n=\frac{Z_{1-\alpha /2}^{2}\times p(1-p)}{d^{2}}$ , where $Z_{1-\alpha /2}$ =1.96, *p* = 0.842, d = 0.05 (23). The estimated sample size was 205. Given the use of non-probability sampling, the sample size was increased by 50%, resulting in a minimum of 308 participants. A larger final sample was targeted to enhance the stability and interpretability of the statistical analysis. At present, there is no universally accepted formula or criterion for determining the required sample size for latent profile analysis (LPA). Rather, the appropriate sample size depends on several factors, including the number of latent profiles to be estimated and the number of indicators included in the model. Evidence from a simulation study suggests that commonly used fit indices for mixture models are generally expected to perform adequately with a minimum sample size of approximately 300 (24).

The inclusion criteria were: (1) Macao residents aged 18 or above; (2) capable of communicating in Chinese (Cantonese or Mandarin); and (3) capable of providing informed consent. Posters and the link to the online survey were distributed to social service organizations and shared on social media platforms (Facebook, WhatsApp, WeChat, etc.). Those who completed the survey were encouraged to share the survey link with eligible Macao residents in their personal networks. For older adults who were unable to complete the online survey independently, trained interviewers administered the questionnaire through face-to-face interviews.

Participant recruitment and data collection were carried out from July through November 2025. This study was approved by the research ethics committee of Kiang Wu Nursing College of Macau (Approval No. REC-2024.0706). All participants were informed about the purpose and procedures of the study, and electronic informed consent was obtained before accessing the questionnaire. All data were collected anonymously and treated confidentially. No identifiable information was recorded. To minimize duplicate submissions, the survey was configured to allow only one response per IP address. After data collection, all responses were manually reviewed for data quality. Responses with obvious response patterns or clear inconsistencies were considered invalid and excluded from the analysis.

### 2.2 Data collection and questionnaire

After reviewing the relevant literature, an online questionnaire was developed based on the TPB framework. In which the “behavioral intention” was operationalized as the willingness to provide bystander resuscitation (20). A panel was then convened to examine the content validity. The experts consisted of (1) a specialist in prehospital and emergency medical care, (2) an associate professor with expertise in prehospital emergency medicine and first aid training, (3) an emergency department physician at a local hospital, (4) a local university lecturer with prior experience as an emergency nurse, and (5) an instructor of the Red Cross, China 120, and the European Resuscitation Council. After revision in response to the expert panel’s suggestions, two waves of cognitive interviews and a pilot test were administered.

Cognitive interviews were conducted to identify wording, phrases, or concepts that might be unclear or open to multiple interpretations (25). After the scale development, two rounds of cognitive interviews were conducted in June 2025, each involving 15 participants. The eligibility criteria were the same as those used in the main survey. To obtain a diverse range of perspectives, participants were purposively recruited through the research team’s personal networks. In the first round, the participants comprised 10 females and 5 males; 7 were aged 18–34 years, 4 were aged 35–54 years, and 4 were aged 55 years or above. In the second round, the participants comprised 7 females and 8 males; 6 were aged 18–34 years, 6 were aged 35–54 years, and 3 were aged 55 years or above. Each participant completed an individual interview conducted by members of the research team. The interviews began with the collection of basic socio-demographic information, followed by concurrent verbal probing (26, 27). Participants were asked to explain their understanding of the item in their own words. When their interpretation differed from the intended meaning, the discrepancy was documented, together with any suggestions for improving the wording. Based on feedback from the first round, relevant items were revised before the second round of cognitive interviews. The cognitive interviews resulted in modifications to wording and readability. Main refinements included adding “under safe conditions” and “after checking” as the situational qualifiers, as well as segmenting lengthy sentences. One item was split into two parallel items by differentiating respondents’ prior training status, addressing trained and untrained laypersons, to more precisely capture attitudinal distinctions based on training background.

Following the cognitive interviews, a pilot test was conducted to evaluate the feasibility of the scale and to further identify alternative expressions that might be more appropriate for the Chinese population. As a minimum sample of 30 participants from the target population is generally recommended for pilot testing (28), 31 participants were recruited. Their ages ranged from 19 to 67 years, with a mean age of 41.42 years (SD = 14.10). There was no revision after the pilot test.

The final version of the questionnaire included 4 subscales: attitude (6 items for BCPR and 6 items for PAD), subjective norm (4 items for BCPR and 4 items for PAD), perceived behavioral control (PBC) (3 items for BCPR and 1 item for PAD), and willingness to provide bystander resuscitation (4 items for BCPR and 1 item for PAD). Each item was rated based on a 5-point Likert scale from 1 (strongly disagree) to 5 (strongly agree). Higher scores indicated a more positive attitude and subjective norm, as well as greater PBC and willingness. Socio-demographic data, such as gender, age, educational level, marital status, employment status, family and personal history of cardiovascular disease, prior resuscitation training, and self-rated health (SRH), were also collected.

### 2.3 Statistical analysis

Descriptive statistics were used to present socio-demographic characteristics, including means and standard deviations (SD) for continuous variables, and frequencies or percentages for categorical variables. Internal consistency of the questionnaire was assessed by Cronbach’s *α* test. To identify the unobserved heterogeneity among individuals with distinct psychosocial profiles of bystander resuscitation, latent profile analysis (LPA) was performed. LPA is a person-centered approach that categorizes unique subgroups (latent profiles) based on the response patterns across a set of continuous indicators. The scores from the six questionnaire subscales, including attitude, subjective norm, and PBC toward BCPR and PAD, were transformed into *z*-scores prior to LPA analysis. We tested models with one to k classes to determine the optimal number of profiles. Model selection was determined using a combination of information criteria, classification quality, and likelihood ratio tests. Lower values of the Akaike information criterion (AIC), Bayesian information criterion (BIC), and sample-size adjusted BIC (aBIC) indicate better model fit. Entropy values over 0.8 indicate acceptable classification precision. Additionally, the Lo–Mendell–Rubin likelihood ratio test (LMR-LRT) and the bootstrapped likelihood ratio test (BLRT) were used to compare the k-profile model with the k-1 profile model. Beyond statistical indices, the final model chosen was also based on parsimony and interpretability. The correlation between socio-demographic characteristics and willingness to provide bystander resuscitation, as well as the differences in socio-demographic characteristics across psychosocial profiles, were examined using chi-square tests. Differences in willingness to provide bystander resuscitation across psychosocial profiles were analyzed using one-way ANOVA, followed by *post hoc* comparisons with the Scheffé or Games-Howell method when appropriate. Multinomial logistic regression was then used to identify socio-demographic factors associated with profile membership. Data management and analyses were performed using IBM SPSS Statistics version 29 (SPSS, Chicago, Illinois, United States). LPA was conducted using Mplus 8.3. The level of significance was set as 0.05.

## 3 Results

### 3.1 Participants’ socio-demographic characteristics

A total of 579 Macao residents completed the online questionnaire, a sample size that exceeded the recommended minimum and was considered sufficient to support stable model estimation. As demonstrated in Table 1, most participants were aged 35 to 54 (*n* = 294, 50.78%), female (*n* = 412, 71.16%), with a higher proportion of an educational level at/or above a Bachelor’s degree (*n* = 428, 73.92%), were married and cohabiting (*n* = 322, 55.61%), employed (*n* = 416, 71.85%), had no family (*n* = 396, 68.39%) and personal (*n* = 545, 94.13%) history of cardiovascular diseases, with prior resuscitation training (*n* = 329, 56.82%), as well as rating not good/very good for self-rating health (*n* = 368, 63.56%). The overall willingness to provide bystander resuscitation score was 18.44 ± 4.09 out of 25.00. Age, education level, prior resuscitation training, and psychosocial profiles were significantly correlated with willingness to provide bystander resuscitation (Table 1).

**Table 1:** Correlation between socio-demographic characteristics and willingness to provide bystander resuscitation (*n* = 579).

Variables	*n*	%	Mean	SD	*F*/*t*	*p*	*Post hoc*
Gender					−1.01	0.315	
Male	167	28.84	18.17	4.27			
Female	412	71.16	18.55	4.01			
Age (years)					14.09^a^	<0.001	1 > 3; 2 > 3
18–34	195	33.68	18.88	3.90			
35–54	294	50.78	18.78	3.87			
> = 55	90	15.54	16.39	4.58			
Education level					−3.06	0.002	
Below bachelor’s degree	151	26.08	17.51	4.50			
Bachelor’s degree or above	428	73.92	18.77	3.88			
Marital status					0.21	0.832	
Not married/separated/divorced/widowed	257	44.39	18.48	4.26			
Married or cohabiting	322	55.61	18.41	3.95			
Employment status					−1.68	0.095	
Not employed^#^	163	28.15	17.94	4.70			
Employed	416	71.85	18.64	3.81			
Family history of cardiovascular diseases					−1.64	0.101	
No	396	68.39	18.25	4.11			
Yes	183	31.61	18.85	4.03			
Personal history of cardiovascular diseases					0.43	0.665	
No	545	94.13	18.46	4.09			
Yes	34	5.87	18.15	4.12			
Prior resuscitation training					−8.10	<0.001	
No	250	43.18	16.92	4.21			
Yes	329	56.82	19.60	3.59			
SRH					−1.77	0.077	
Not good/very good	368	63.56	18.21	4.01			
Good/very good	211	36.44	18.84	4.19			
Psychosocial profiles					59.83^b^	<0.001	1 < 2; 1 < 3; 1 < 4 2 > 3; 2 < 4 3 < 4
Profile 1: Reluctant	138	23.83	15.89	3.95			
Profile 2: Socially inhibited	162	27.98	19.04	3.48			
Profile 3: Technically constrained	146	25.22	17.45	4.04			
Profile 4: Proactive	133	22.97	21.44	2.61			

^a^(*p* > 0.05) homogeneity of variance was assumed. The Scheffé method was used for *post hoc* comparisons.

^b^(*p* < 0.05) homogeneity of variance was not assumed. The Games–Howell method was used for *post hoc* comparisons.

SD, standard deviation.

SRH, self-rated health.

^#^ includes housewife/househusband, unemployed, retired, student.

### 3.2 Validity and reliability of the questionnaires

The questionnaire subscales demonstrated varying levels of internal consistency, with Cronbach’s *α* values of 0.528 for attitude (BCPR), 0.619 for attitude (PAD), 0.707 for subjective norm (BCPR), 0.750 for subjective norm (PAD), 0.848 for PBC (BCPR), and 0.847 for willingness to provide bystander resuscitation. Given that the PBC (PAD) was operationalized using a single item, the Cronbach’s *α* value was not applied. Although the Cronbach’s α values for the attitude (BCPR) and attitude (PAD) subscales were relatively low, these subscales were retained because attitude is a core construct of the TPB framework, and the items were developed based on relevant literature, reviewed by an expert panel, and supported by acceptable content validity. Given that the content validity index of the subscales ranged from 0.80 to 1.00, the attitude subscales were considered theoretically and substantively relevant for inclusion in the LPA.

### 3.3 Model selection

Although the AIC, BIC, and aBIC values continued to decrease from the four-profile model to the five- and six-profile models, the four-profile solution was selected as the final model based on overall model evaluation rather than information criteria alone. Specifically, the four-profile model demonstrated acceptable classification quality, as indicated by an entropy value above 0.80, and showed significant improvement over the three-profile model according to both the BLRT and LMR-LRT (*p* < 0.001). In contrast, the five- and six-profile models did not yield significant BLRT or LMR-LRT values, suggesting that the additional profiles did not provide meaningful improvement in model fit. The four-profile solution was also preferred because it offered greater parsimony and clear interpretability (Table 2). Therefore, all subsequent analyses were conducted using the four-profile model.

**Table 2:** Model fit indices and selection criteria for LPA with 1 to 6 profiles (*n* = 579).

Model	AIC	BIC	aBIC	Entropy	*p* for BLRT	*p* for LMR
1-Profile	9876.78	9929.12	9891.02	–	–	–
2-Profile	9009.72	9092.59	9032.27	0.80	0.002	0.003
3-Profile	8795.76	8909.15	8826.61	0.82	0.011	0.012
4-Profile	8509.87	8653.79	8549.03	0.83	<0.001	<0.001
5-Profile	8410.24	8584.70	8457.71	0.84	0.169	0.174
6-Profile	8308.30	8513.28	8364.07	0.85	0.083	0.087

AIC, Akaike information criterion; BIC, Bayesian information criterion; aBIC, Sample size adjusted BIC; BLRT, Bootstrap likelihood ratio test; LMR, Lo–Mendell–Rubin adjusted likelihood ratio.

### 3.4 Psychosocial profiles of bystander resuscitation

**Figure 1:**
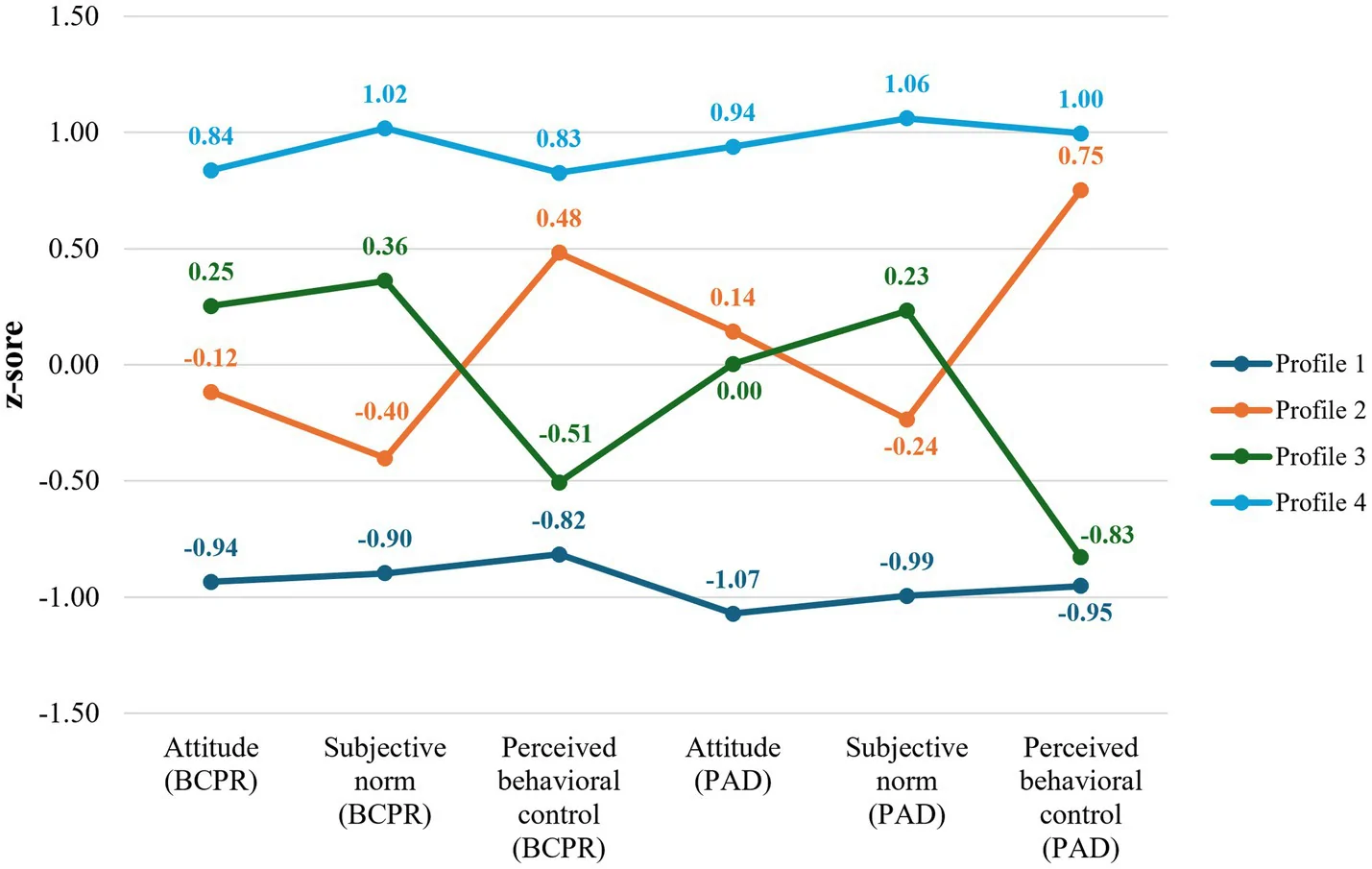
The *z*-score of each TPB construct within psychosocial profiles.

The four psychosocial profiles of bystander resuscitation, differentiated by the *z*-scores of the TPB constructs, were illustrated in Figure 1. The socio-demographic characteristics across the four profiles were presented in Table 3. The distribution patterns of *z*-scores across constructs for BCPR were nearly identical to those observed for PAD in all psychosocial profiles. Profile 1 (reluctant) comprised 23.83% (*n* = 138) of respondents. Members in this profile exhibited uniformly lowest levels across all TPB constructs, with z-scores ranging from −0.82 to −1.07 (BCPR: −0.82 to −0.94; PAD: −0.95 to −1.07). This profile was characterized by the lowest levels of attitudes, subjective norms, and PBC toward both BCPR and PAD. Profile 2 (socially inhibited) comprised 27.98% (*n* = 162) of respondents. Members in this profile demonstrated the second-highest PBC (BCPR *z*-score: 0.48; PAD *z*-score: 0.75) and the second-lowest subjective norms (BCPR: −0.40 *z*-scores; PAD: −0.24 *z*-scores). Notably, this profile exhibited a divergent pattern in attitudes, with a below-average attitude toward BCPR (*z*-score: −0.12) and above-average attitude toward PAD (*z*-score: 0.14). Profile 3 (technically constrained) comprised 25.22% (*n* = 146) of respondents. Members in this profile represented the second-lowest PBC (BCPR *z*-score: −0.51, PAD *z*-score: −0.83) but the second-highest attitude (*z*-score: 0.25) and subjective norm (*z*-score: 0.36) toward BCPR, while nearly at an average level of attitude (*z*-score: 0.003) and the second-highest subjective norm (*z*-score: 0.23) toward PAD. Profile 4 (proactive) comprised 22.97% (*n* = 133) of all respondents. Members of this profile demonstrated consistently the highest levels across all TPB constructs related to BCPR and PAD, with *z*-scores ranging from 0.83 to 1.06.

**Table 3:** Socio-demographic characteristics across psychosocial profiles.

Variables	Profile 1		Profile 2		Profile 3		Profile 4		*X* ^2^	*p*
	*n*	%	*n*	%	*n*	%	*n*	%		
Gender									1.29	0.731
Male	40	28.99%	50	30.86%	37	25.34%	40	30.08%		
Female	98	71.01%	112	69.14%	109	74.66%	93	69.92%		
Age (years)									52.86	<0.001
18–34	45	32.61%	68	41.98%	23	15.75%	59	44.36%		
35–54	71	51.45%	80	49.38%	79	54.11%	64	48.12%		
> = 55	22	15.94%	14	8.64%	44	30.14%	10	7.52%		
Education level									14.77	0.002
Below bachelor’s degree	32	23.19%	39	24.07%	55	37.67%	25	18.80%		
Bachelor’s degree or above	106	76.81%	123	75.93%	91	62.33%	108	81.20%		
Marital status									6.96	0.073
Not married/separated/divorced/widowed	62	44.93%	83	51.23%	53	36.30%	59	44.36%		
Married or cohabiting	76	55.07%	79	48.77%	93	63.70%	74	55.64%		
Employment status									10.57	0.014
Not employed^#^	40	28.99%	39	24.07%	55	37.67%	29	21.80%		
Employed	98	71.01%	123	75.93%	91	62.33%	104	78.20%		
Family history of cardiovascular diseases									2.16	0.540
No	95	68.84%	109	67.28%	106	72.60%	86	64.66%		
Yes	43	31.16%	53	32.72%	40	27.40%	47	35.34%		
Personal history of cardiovascular diseases									3.56	0.313
No	129	93.48%	153	94.44%	134	91.78%	129	96.99%		
Yes	9	6.52%	9	5.56%	12	8.22%	4	3.01%		
Prior resuscitation training									122.52	<0.001
No	95	68.84%	39	24.07%	93	63.70%	23	17.29%		
Yes	43	31.16%	123	75.93%	53	36.30%	110	82.71%		
SRH									7.22	0.065
Not good/very good	96	69.57%	95	58.64%	100	68.49%	77	57.89%		
Good/very good	42	30.43%	67	41.36%	46	31.51%	56	42.11%		

^#^ includes housewife/househusband, unemployed, retired, student.

SRH, self-rated health.

Results of one-way ANOVA (Table 1) revealed significant differences in willingness to provide bystander resuscitation across the four profiles (*F* = 59.83, *p* < 0.001). *Post hoc* tests indicated that Profile 4 demonstrated the highest willingness to provide bystander resuscitation (*M* = 21.44, SD = 2.61), significantly exceeding those of all other profiles (all *p* < 0.001). Profile 2 (*M* = 19.04, SD = 3.48) demonstrated significantly higher willingness than both Profile 3 (*M* = 17.45, SD = 4.04; *Δ* = 1.59, *p* = 0.002) and Profile 1 (*M* = 15.89, SD = 3.95; mean Δ = 3.15, *p* < 0.001), whereas willingness of Profile 3 significantly higher than Profile 1 (Δ = 1.56, *p* = 0.006). Significant differences in socio-demographic characteristics, such as age, educational level, employment status, and prior resuscitation training, were observed across the four profiles, as shown in Table 3.

### 3.5 Factors associated with profile membership

A multinomial logistic regression model was used to identify factors associated with profile membership, with Profile 4 (proactive) as the reference. The results in Table 4 indicated that prior resuscitation training and age were the independent factors associated with profile membership. Specifically, individuals who lacked prior resuscitation training were significantly more likely to be classified into Profile 1 (reluctant) (OR = 10.84, 95% CI [5.93, 19.82], *p* < 0.001) and Profile 3 (technically constrained) (OR = 6.71, 95% CI [3.69, 12.19], *p* < 0.001) rather than Profile 4 (proactive). In addition, age was also a significant associated factor for Profile 3. Middle-aged (aged 35–54) (OR = 2.91, 95% CI [1.50, 5.64], *p* = 0.002) and older-aged (aged > 55) (OR = 4.75, 95% CI [1.80, 12.52], *p* = 0.002) individuals were significantly more likely to be classified into Profile 3 compared to the younger reference group (aged 18–34). Other socio-demographic factors did not significantly differentiate the psychosocial profiles.

**Table 4:** Socio-demographic factors associated with profile membership (Profile 4 as reference group).

Profile	Variables^a^	*B*	Std. error	Wald	*p*	Exp(B)	95% Confidence Interval for Exp(B)	
							Lower bound	Upper bound
1	Intercept	−1.00	0.78	1.66	0.197			
	Male	−0.41	0.30	1.93	0.165	0.66	0.37	1.19
	Aged > = 55	0.37	0.51	0.53	0.466	1.45	0.53	3.96
	Aged 35–54	0.35	0.32	1.21	0.271	1.41	0.76	2.62
	Below bachelor’s degree	−0.15	0.37	0.16	0.685	0.86	0.42	1.78
	Not married/separated/divorced/widowed	0.24	0.30	0.67	0.413	1.27	0.71	2.28
	Not employed^#^	−0.12	0.36	0.11	0.738	0.89	0.44	1.80
	No family history of cardiovascular diseases	0.31	0.29	1.18	0.277	1.36	0.78	2.38
	No personal history of cardiovascular diseases	−0.54	0.67	0.64	0.423	0.59	0.16	2.17
	Without prior resuscitation training	2.38	0.31	59.91	<0.001	10.84	5.93	19.82
	SRH: not good/very good	0.34	0.28	1.45	0.228	1.40	0.81	2.42
2	Intercept	0.29	0.70	0.17	0.678			
	Male	−0.02	0.26	0.01	0.936	0.98	0.59	1.64
	Aged > = 55	0.02	0.49	0.00	0.975	1.02	0.39	2.65
	Aged 35–54	0.20	0.27	0.54	0.462	1.22	0.72	2.08
	Below bachelor’s degree	0.25	0.33	0.56	0.455	1.28	0.67	2.45
	Not married/separated/divorced/widowed	0.35	0.26	1.74	0.187	1.41	0.85	2.36
	Not employed^#^	−0.08	0.33	0.06	0.800	0.92	0.48	1.75
	No family history of cardiovascular diseases	0.16	0.25	0.41	0.521	1.18	0.72	1.92
	No personal history of cardiovascular diseases	−0.60	0.62	0.92	0.339	0.55	0.16	1.87
	Without prior resuscitation training	0.41	0.31	1.83	0.176	1.51	0.83	2.75
	SRH (not good/very good)	−0.01	0.24	0.00	0.985	1.00	0.62	1.60
3	Intercept	−1.52	0.77	3.84	0.050			
	Male	−0.37	0.30	1.53	0.217	0.69	0.39	1.24
	Aged > = 55	1.56	0.50	9.90	0.002	4.75	1.80	12.52
	Aged 35–54	1.07	0.34	9.93	0.002	2.91	1.50	5.64
	Below bachelor’s degree	0.32	0.35	0.83	0.362	1.37	0.69	2.72
	Not married/separated/divorced/widowed	−0.05	0.30	0.03	0.868	0.95	0.53	1.70
	Not employed^#^	0.01	0.36	0.00	0.988	1.01	0.50	2.03
	No family history of cardiovascular diseases	0.50	0.29	2.99	0.084	1.64	0.94	2.88
	No personal history of cardiovascular diseases	−0.45	0.65	0.49	0.485	0.64	0.18	2.27
	Without prior resuscitation training	1.90	0.31	39.00	<0.001	6.71	3.69	12.19
	SRH (not good/very good)	0.26	0.28	0.89	0.345	1.30	0.76	2.23

^#^ includes housewife/ househusband, unemployed, retired, student.

^a^ reference for the variables: female, aged 18–34, bachelor’s degree or above, married or cohabiting, employed, with family history of cardiovascular disease, with personal history of cardiovascular diseases, with prior resuscitation training and SRH (good/very good).

SRH, self-rated health.

## 4 Discussion

The person-centered LPA revealed that TPB constructs could operate synergistically, manifesting as specific configurational patterns across psychosocial profiles. In which, the remarkably consistent patterns for both BCPR and PAD within each profile suggested that individuals’ psychological tendency toward these two resuscitation behaviors was highly synchronized. Prior resuscitation training and age appeared to be independent factors that differentiated the psychosocial profiles of bystander resuscitation, underscoring their potential role in influencing willingness to provide bystander resuscitation.

Few studies have applied LPA to investigate how different psychosocial profiles associate with willingness to provide bystander resuscitation. Yang et al. (29) adopted eight CPR-related indicators, such as “call emergency services,” “perform chest compression,” “administer rescue breathing,” etc., to classify participants into low-, medium-, and high-behavior groups using LPA. The authors found that the low behavioral group was associated with low self-efficacy, an indicator of PBC. However, our study clustered individuals based on core TPB constructs rather than focusing on discrete BCPR and PAD actions. We also elucidated how these constructs interact within individuals. Profiles 4 (proactive) and 1 (reluctant) represented two ends of the spectrum. When positive attitudes, strong subjective norms, and higher PBC were combined, as in Profile 4, resulting in the highest willingness to provide bystander resuscitation. In contrast, with an opposite combination of those factors, Profile 1 presented the lowest willingness. Consistent with previous variable-centered studies, attitudes, subjective norms, and PBC were significantly associated with willingness to provide BCPR and PAD across various populations. Tam and Kwok (20) found that these factors all positively influenced the willingness to provide BCPR and PAD (all aORs > 1.5) among community residents. Similar findings were reported in lay community participants (30), civil servants (31), and university students (32, 33). This finding suggested that when TPB constructs exhibited directional consistency, with either uniformly high or uniformly low, it might strengthen their overall prediction of willingness to provide bystander resuscitation.

On the other hand, Profiles 2 (socially inhibited) and 3 (technically constrained) demonstrated another interactive pattern among TPB constructs. Profile 2 presented notably low subjective norms toward BCPR and PAD. The low level of subjective norm might be attributed to several sociocultural factors. Unlike many Western countries, Macao has not enacted the “Good Samaritan Law” or related Acts to protect lay persons from potential civil liability when providing BCPR or PAD (34). This legislative gap intensifies bystanders’ fear of litigation and legal liability (35, 36). It may also influence the normative beliefs held by significant others regarding whether laypersons should intervene in an OHCA event, thereby weakening the subjective norm component of the TPB (37). In addition, recent epidemiological data indicated that the average EMS response time for OHCA in Macao was 3.2 min (38). The rapid arrival of professional medical personnel may further reduce societal expectations for laypersons to initiate BCPR and PAD. Our studies found that the substantially high PBC in Profile 2 effectively counterbalanced this disadvantage, resulting in the second-highest willingness to provide bystander resuscitation. In contrast, the markedly lower PBC in Profile 3 diminished the effects of moderately high subjective norms and attitudes toward BCPR and PAD, thereby leading to the second-lowest willingness. As suggested by La Barbera and Ajzen (39), the higher level of PBC can enhance the relative contribution of attitude to intention (willingness) while attenuating the influence of subjective norm. Taken together, these configurational patterns suggest that PBC may play a compensatory role in the interplay among attitudes, subjective norms, and willingness to provide bystander resuscitation. However, this interpretation warrants further investigation through moderating analysis.

The results of MLR demonstrated that, lacking prior resuscitation training, individuals had approximately a 10-fold and a 6-fold higher likelihood of being classified as Profile 1 (reluctant) and Profile 3 (technically constrained), respectively. A systematic review concluded that training within the past 5 years and participating in 4 or more sessions were determinants of increased willingness to provide BCPR and PAD (9). Empirical studies also demonstrated that training significantly improved participants’ PBC to perform CPR and use an AED (40–42), which is particularly important for individuals in those Profiles exhibiting markedly low PBC. Regarding age, respondents aged 35 and over were more likely to be classified as Profile 3, a group with the second-lowest willingness to provide bystander resuscitation. This finding was opposite to existing evidence that individuals aged 40 and over were associated with greater willingness (31, 43). Profile 3 exhibited relatively high attitudes and subjective norms toward BCPR and PAD, potentially reflecting greater empathic and prosocial tendencies with aging (44, 45). The low willingness might be mainly attributed to the substantially low PBC (46, 47). Therefore, resuscitation training programs should be tailored to enhance PBC and to account for different age groups.

From a practical perspective, these distinct psychosocial profiles of bystander resuscitation, together with factors associated with profile membership, informed the design of targeted strategies to increase willingness to provide bystander resuscitation in the Macao community. For Profile 1 (reluctant), characterized by globally low attitudes, subjective norms, and PBC, a comprehensive approach is warranted, combining public awareness campaigns to improve attitudes, community-based reinforcement of social norms, and structured CPR/AED training to strengthen PBC toward BCPR and PAD (30). For Profile 2 (socially inhibited), who showed high PBC but notably low subjective norms, interventions might prioritize legal reassurance (48) and social-norm reinforcement strategies, such as mass media campaigns that normalize BCPR and PAD. For Profile 3 (technically constrained), who had relatively positive attitudes and subjective norms but markedly low PBC, skill-oriented training with simulation-based learning and virtual reality training might be most beneficial (49). For Profile 4 (proactive), who already demonstrated high levels across all constructs, efforts should focus on sustaining engagement through refresher training (9). Finally, with the global population aging, the incidence of OHCA among older adults has increased (50). Older adults are increasingly likely to encounter OHCA in community or home settings (36). Given that older adults frequently experience age-related physical limitations and cognitive decline, a specific resuscitation training program should be simplified and tailored to their functional capacities, such as compression-only CPR (51, 52). However, modified AED training for older adults remains lacking.

To the best of our knowledge, this is among the first studies using a person-centered analytic approach to investigate psychosocial profiles of bystander resuscitation within the TPB framework in an Asian community. This approach offered a new angle for examining how attitudes, subjective norms, and PBC interact at the individual level, providing a refined framework for developing profile-specific interventions. However, several limitations needed to be addressed. First, a cross-sectional design precluded causal inferences regarding the relationship among factors associated with profile membership. Second, self-reported questionnaires may introduce recall and social desirability biases, potentially inflating willingness to provide bystander resuscitation. Third, from the perspective of the TPB scales, the relatively low Cronbach’s *α* values for the attitude (BCPR) and attitude (PAD) subscales indicated limited internal consistency. Supplementary exploratory factor analyses (Supplementary Tables S1, S2) revealed a two-factor structure underlying the attitude subscales for both BCPR and PAD, comprising beliefs about the feasibility of BCPR/PAD under specific conditions and beliefs concerning the perceived importance of BCPR/PAD, professional role boundaries, and personal responsibility. This bifurcation structure is consistent with Ajzen’s (53) observation that behavioral beliefs may simultaneously connect a behavior to both positive and negative outcomes, leading to attitudinal ambivalence and lower internal consistency. Given that Ajzen’s theory is not specifically designed to explain willingness to provide bystander resuscitation, our findings may inform refinement of the “intention-focused model for bystander” advocated by the American Heart Association, which adopts Ajzen’s TPB as its theoretical basis (36). On the other hand, although PBC toward PAD was measured with a single item, it was conceptually appropriate. Unlike BCPR, which involves a sequence of skills (i.e., assessing consciousness, performing chest compressions, and giving rescue breaths), PAD is a specific skill and can be captured by a self-rated level of capacity to operate an AED. Single-item measures of self-rated status or behaviors have demonstrated acceptable validity in health behavior research and reduce respondent burden in survey-based studies (54). However, we acknowledged that the external factors of the PBC, such as perceived availability of AED devices in public areas, were not captured. Collectively, future studies are encouraged to examine the effects of different attitude-related factors on willingness to provide bystander resuscitation, and to develop a more comprehensive assessment of PBC toward PAD to improve internal consistency and generalizability. Fourth, the use of convenience and snowball sampling may limit the representativeness and generalizability of the findings to represent Macao residents. Non-probability sampling techniques are inherently susceptible to selection bias, as individuals who are more accessible and socially connected are more likely to participate. Fifth, prior Chinese studies reported that males and those with lower education levels exhibited a higher willingness to provide BCPR and awareness of PAD (43), whereas males and individuals with higher education levels tended to demonstrate more positive subjective norms and higher PBC toward BCPR (31). In addition, prior resuscitation training was more prevalent among individuals with higher levels of education (55). Given that females and higher-educated participants were overrepresented in our study, the observed willingness, profile distribution, and prior training may have been influenced accordingly. Finally, willingness to provide bystander resuscitation, as a proxy for actual bystander behavior, may not fully capture real-world behavioral responses. Therefore, the findings should be interpreted with appropriate caution.

## 5 Conclusion

By integrating the TPB with LPA, this study identified four distinct psychosocial profiles of bystander resuscitation among Macao residents, offering a nuanced understanding of population heterogeneity in the willingness and psychological preparedness to provide bystander resuscitation during OHCA. These findings reflect behavioral orientations rather than confirmed real-world resuscitation actions. Understanding the factors associated with profile membership can further inform training providers and policymakers in developing more targeted interventions, including profile- and age-specific CPR and AED training programs, while also promoting public awareness and social acceptance of bystander resuscitation to enhance community preparedness. Longitudinal or intervention-based designs may be conducted in future studies to clarify causal pathways among resuscitation training, PBC, and transitions across psychosocial profiles.

## Data Availability

The datasets presented in this article are not readily available because the data supporting the findings of this study are available from the corresponding author upon reasonable request. Requests to access the datasets should be directed to Sok Leng Che, shirley@kwnc.edu.mo.
