# Supplementary material for: Psychosocial profiles of bystander resuscitation among Macao residents: a latent profile analysis
Source: Front Public Health. 2026 Aug 7;14:1867128. doi: 10.3389/fpubh.2026.1867128 (PMC13493374; doi:10.3389/fpubh.2026.1867128)
Supplement: Supplementary file 1 [file data_sheet_1.pdf]

Supplementary Table

Table S1. Pattern matrix of exploratory factor analysis for the attitude (BCPR) subscale (n=579)

Item	Factor 1	Factor 2
Laypersons without CPR training can perform CPR under safe conditions	**0.828**	− 0.244
Laypersons without CPR training can perform CPR under remote professional guidance	**0.714**	0.18
Laypersons with CPR training can perform CPR under safe conditions	**0.593**	0.401
Performing CPR on a stranger in cardiac arrest is none of my business	− 0.351	**0.781**
CPR should only be performed by professional rescue or medical personnel	0.147	**0.621**
CPR is an important life-saving resuscitative intervention	0.159	**0.561**

Note. Extraction method: principal component analysis. Rotation method: oblimin with Kaiser normalization.

Table S2. Pattern matrix of exploratory factor analysis for the attitude (PAD) subscale

Item	Factor 1	Factor 2
Laypersons without AED training can use an AED under remote professional guidance	**0.827**	0.031
Laypersons without AED training can use an AED under safe conditions	**0.786**	−0.218
Laypersons with AED training can use an AED under safe conditions	**0.671**	0.206
Using an AED on a stranger in cardiac arrest is none of my business	−0.256	**0.915**
An AED should only be used and operated by professional rescue or medical personnel	0.294	**0.564**
Using an AED on a person in cardiac arrest is an important life-saving resuscitative intervention	0.290	**0.348**

Note. Extraction method: principal component analysis. Rotation method: oblimin with Kaiser normalization.
